# Automated Coronary Artery Calcification Scoring in Non-Gated Chest CT: Agreement and Reliability

**DOI:** 10.1371/journal.pone.0091239

**Published:** 2014-03-13

**Authors:** Richard A. P. Takx, Pim A. de Jong, Tim Leiner, Matthijs Oudkerk, Harry J. de Koning, Christian P. Mol, Max A. Viergever, Ivana Išgum

**Affiliations:** 1 Department of Radiology, University Medical Center Utrecht, Utrecht, The Netherlands; 2 Center for Medical Imaging – North East, Netherlands, University Medical Center Groningen, University of Groningen, Groningen, The Netherlands; 3 Department of Public Health, Erasmus Medical Center Rotterdam, Rotterdam, The Netherlands; 4 Images Sciences Institute, University Medical Center Utrecht, Utrecht, The Netherlands; University of Navarra, Spain

## Abstract

**Objective:**

To determine the agreement and reliability of fully automated coronary artery calcium (CAC) scoring in a lung cancer screening population.

**Materials and Methods:**

1793 low-dose chest CT scans were analyzed (non-contrast-enhanced, non-gated). To establish the reference standard for CAC, first automated calcium scoring was performed using a preliminary version of a method employing coronary calcium atlas and machine learning approach. Thereafter, each scan was inspected by one of four trained raters. When needed, the raters corrected initially automaticity-identified results. In addition, an independent observer subsequently inspected manually corrected results and discarded scans with gross segmentation errors. Subsequently, fully automatic coronary calcium scoring was performed. Agatston score, CAC volume and number of calcifications were computed. Agreement was determined by calculating proportion of agreement and examining Bland-Altman plots. Reliability was determined by calculating linearly weighted kappa (κ) for Agatston strata and intraclass correlation coefficient (ICC) for continuous values.

**Results:**

44 (2.5%) scans were excluded due to metal artifacts or gross segmentation errors. In the remaining 1749 scans, median Agatston score was 39.6 (P25–P75∶0–345.9), median volume score was 60.4 mm^3^ (P25–P75∶0–361.4) and median number of calcifications was 2 (P25–P75∶0–4) for the automated scores. The κ demonstrated very good reliability (0.85) for Agatston risk categories between the automated and reference scores. The Bland-Altman plots showed underestimation of calcium score values by automated quantification. Median difference was 2.5 (p25–p75∶0.0–53.2) for Agatston score, 7.6 (p25–p75∶0.0–94.4) for CAC volume and 1 (p25–p75∶0–5) for number of calcifications. The ICC was very good for Agatston score (0.90), very good for calcium volume (0.88) and good for number of calcifications (0.64).

**Discussion:**

Fully automated coronary calcium scoring in a lung cancer screening setting is feasible with acceptable reliability and agreement despite an underestimation of the amount of calcium when compared to reference scores.

## Introduction

Smoking is an important factor in the etiology of cardiovascular disease (CVD) [1], [2]. Coronary artery calcification (CAC) is observed frequently in patients with cardiovascular events and in advanced atherosclerotic plaques [3]. CAC scoring with ECG-gated computed tomography (CT) has emerged as an important imaging biomarker for CVD and all-cause mortality [4], [5], [6]. Based on CAC scores, patients can be assigned into CVD risk categories to guide treatment [7].

Low-dose non-gated chest CT has been applied for lung cancer screening in smokers [8], [9]. In spite of suboptimal image acquisition, CAC scoring from lung cancer screening CT has been shown to be a strong and independent predictor of cardiovascular events and all-cause mortality [10], [11], [12]. Also, several studies demonstrated good agreement between CAC scores determined using low-dose non-gated CT, as acquired in lung cancer screening, and CAC scores quantified using gated cardiac CT [13], [14], [15]. Budoff et al. [14] and Kim at al. [15] found a correlation of 0.96 and 0.89 between Agatston CAC scores obtained with and without gated CT, respectively. Furthermore, intraclass correlation coefficient (ICC) between absolute Agatston scores on two low-dose ungated CT scans within four months was very good (0.94) [16]. These findings indicate that CAC scores obtained in lung cancer screening setting can be used for identification of subjects at risk of CVD events. Integrated screening for lung cancer and CVD in smokers could optimize risk prediction without additional radiation exposure for the participant. Manual scoring of CAC on low-dose non-gated CT is time-consuming as a result of the increased number of slices and the high prevalence of coronary calcification, difficult due to cardiac motion and thus cumbersome and expensive in a screening setting. Moreover, manual scoring may add to inter-rater variability, although a previous study found an ICC between human raters of 0.97 in a small set of 50 randomly selected CT scans [16]. Automated quantification of CAC could overcome these limitations and previous studies demonstrated preliminary feasibility using non-gated CT [17].

The objective of our study was to determine the agreement and reliability of automated CAC scoring compared with reference scores in a large set of scans acquired in a lung cancer screening data.

## Methods

### Participants

This study included participants of lung cancer screening trial who smoked 15 or more cigarettes per day for 25 years or 10 or more cigarettes for 30 years, and were current smoker or had quit less than 10 years ago.

### Data

This current study is an ancillary study of the Dutch-Belgian Randomized Lung Cancer Screening Trial (Dutch acronym: NELSON study) (ISRCTN63545820) and was approved by the institutional ethical boards of the participating medical centers (University Medical Centre Groningen, University Medical Centre Utrecht, Kennemer Gasthuis Haarlem [the Netherlands], and University Hospital Leuven [Belgium]). Furthermore, the Ministery of Health approved the NELSON trial after positive advice of the Dutch Health Council. Written informed consent was obtained from all participants. The NELSON study was designed to investigate whether lung cancer screening by low-dose CT will reduce 10-year lung cancer mortality by at least 25% in high-risk (ex-)smokers between ages 50 and 75 compared with a control group without screening.

### Computed Tomography

Images were obtained in University Medical Center Utrecht on a 16-slice CT scanner with a 16 mmx0.75 mm collimation (Mx8000 IDT or Brilliance-16P CT, Philips Medical Systems, Best, the Netherlands). A 120 kV tube voltage was applied in participants weighing less than 80 kg and in participants weighing more than 80 kg the tube voltage was increased to 140 KV. The mAs settings were depended on the CT hardware used and adjusted accordingly. All scans were reconstructed to a slice thickness of 3.1 mm and an increment of 1.4 mm [18].

### Reference Standard

Manual CAC scoring in chest CT scans from lung cancer screening study is extremely time-consuming and cumbersome due to cardiac motion, image noise and numerous calcifications in high-risk population [17]. Hence, to set the reference standard that enables evaluation of the automatic method in a large data set from this study the following approach was utilized. First, coronary calcifications were identified automatically, using the preliminary version of the evaluated algorithm for automated CAC scoring [19]. Thereafter, four trained raters, a radiologist with six years of experience in cardiac CT and three medical students, set the reference standard for this study. The raters inspected and when deemed necessary corrected the errors of the algorithm. Each scan was inspected by one of the four raters. Prior to this, the medical students received extensive training (e.g. reviewed at least 100 scans) for this study by a board certified chest radiologist. Readers were blinded to the participant’s age, sex and clinical data. Visually identified stents were excluded from quantification. Also, the raters discarded scans with artifacts caused by metal implants. Finally, to ensure high quality of the reference standard, one research physician with four years of experience in cardiac CT evaluated all cases and excluded those containing gross segmentation errors, i.e. incorrectly identified lesions as coronary calcifications, or coronary calcifications missed by the raters. In such a way identified coronary calcifications served as a reference study for further evaluation.

### Automated Quantification

CAC scores were automatically quantified without any user interaction using previously published algorithm [17]. The software applied a threshold of 130 HU in combination with three-dimensional connected component labeling to mark potential calcifications (candidates). Subsequently, each candidate was described by size, spatial and texture characteristics. Volume of each candidate was used a size feature. Spatial features were determined using a coronary calcium atlas providing an a priori probability for spatial appearance of coronary calcifications in a chest CT scan (e.g. spatial probability that a candidate is a coronary calcification). Texture features were computed using Gaussian filters at multiple scales. Based on the features, coronary calcifications were identified using a supervised pattern recognition system with k-nearest neighbor and support vector machine classifiers. Finally, identified coronary calcifications were quantified as Agatston score and total calcium volume (mm^3^). To determine CVD risk of subjects, Agatston score was divided into five strata (0, 1–10, 11–100, 101–400, and >400) [20].

### Manual Measurements

To determine human interrater reliability and to establish whether presegmentation of coronary calcifications by automatic software, i.e. initial automatic identification of coronary artery calcifications, influenced the reference scores, the same four raters independently scored a subset of 199 consecutive CT scans fully manually, thus without any presegmentation.

### Statistical Analysis

Normally distributed data are presented as mean ± standard deviation (SD) and non-normally distributed data as median plus 25th–75th percentile (P25–P75). Quartile coefficient of dispersion (QCD) was calculated to determine dispersion. Inter-rater agreement and reliability were calculated [21], [22]. Agreement is the degree to which the scores are identical and reliability is defined as the ratio of variability between CT scans to the total variability of all quantifications in the sample. Agreement is especially important when assessing the usability of a score to monitor health status-changes over time using repeated measurements. Agreement was determined by calculating the proportion of subjects with the same CVD risk determined by the reference and automatically, and examining Bland-Altman plots with 95% limits of agreement. The measurement error of CAC score increases with higher CAC scores [23]. Accordingly, we applied a regression approach for non-uniform differences to model the variation of the absolute differences between the two measurement techniques [24]. The 95% repeatability limits were calculated by multiplying the predicted absolute difference by 1.96×(π/2)^0.5^, since the absolute difference has a half-normal distribution [25]. Reliability is the degree to which the test can effectively distinguish between study participants, regardless of rater error. Reliability is of importance in diagnostic practice to distinguish between affected and non-affected persons at a single time-point. Reliability between automated and reference quantification and between fully manual scoring and reference scoring was determined by calculating linearly weighted kappa (κ) for Agatston strata and two-way-mixed ICC for continuous values. Interrater reliability of fully manual scoring was calculated using Kendall’s coefficient of concordance (Kendall’s w) for Agatston risk categories and two-way-random ICC for continuous values. P values <0.05 were considered to be statistically significant. Statistical analyses were performed with SPPS version 19 (SPSS Inc, Chicago, Illinois, USA) and R version 2.10.2 (R Foundation for Statistical Computing, Vienna, Austria).

## Results

1793 participants (median age 60.1, P25–P75 56.7–64.3 years; 97 females) underwent a non-contrast enhanced non-ECG-gated CT of the chest. 44 scans were discarded because of beam hardening artifacts (18), or gross segmentation errors (26). Median Agatston score was 55.8 (P25–P75∶1.1–449.0; QCD: 1.00; range: 0–12080.9), median volume score was 87.4 mm^3^ (P25–P75∶3.2–509.7; QCD: 0.99; range: 0–9610,9) and median number of calcifications was 3 (P25–P75∶1–9; QCD: 0.80; range: 0–53) based on the reference scores; and 39.6 (P25–P75∶0–345.9; QCD: 1.00; range: 0–8363.3), 60.4 mm^3^ (P25–P75∶0–361.4; QCD: 1.00; range:0–6656.1) and 2 (P25–P75∶0–4; QCD: 1.00; range: 0–35) based on automated scores, respectively.

### Agreement between Reference and Automated Cac Score

**Figure 1:**
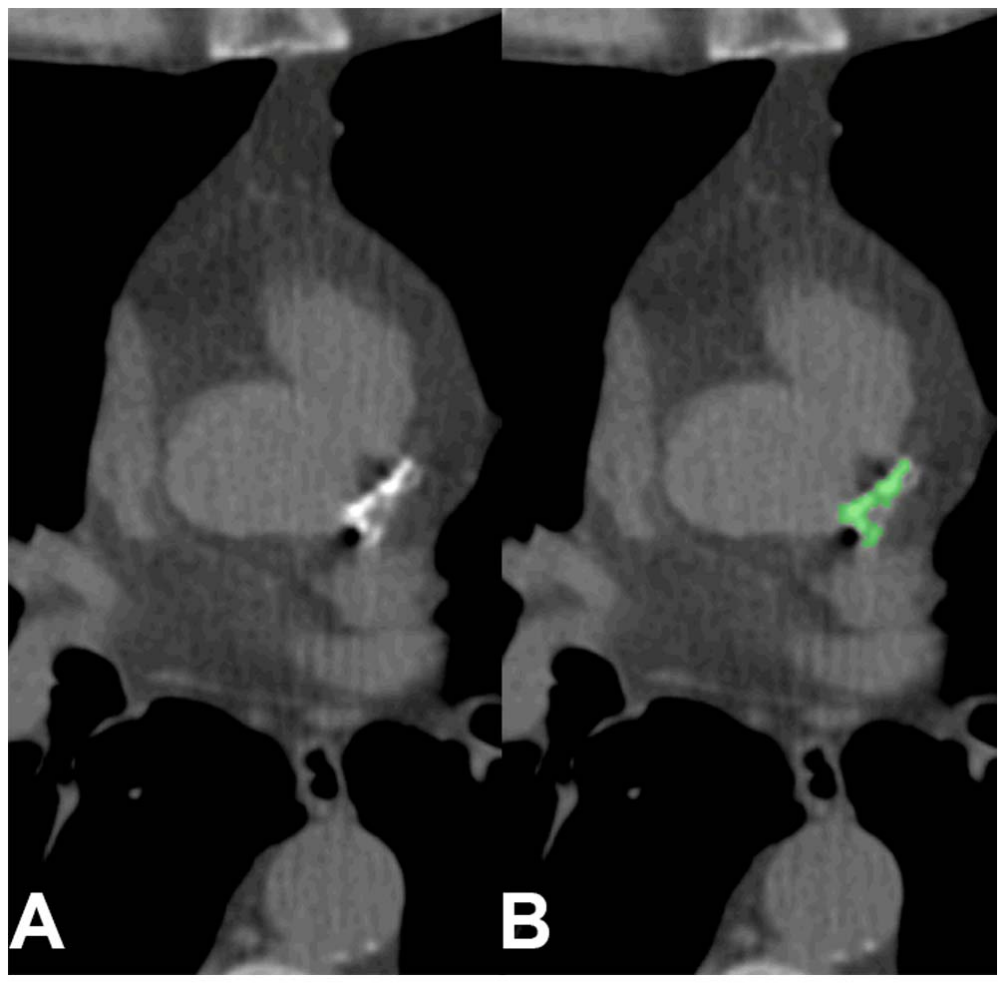
False negative by automated scoring. Example of missed calcification in the LM by automated scoring method (A) compared with reference calcification in green (B). No stent was present.

**Figure 2:**
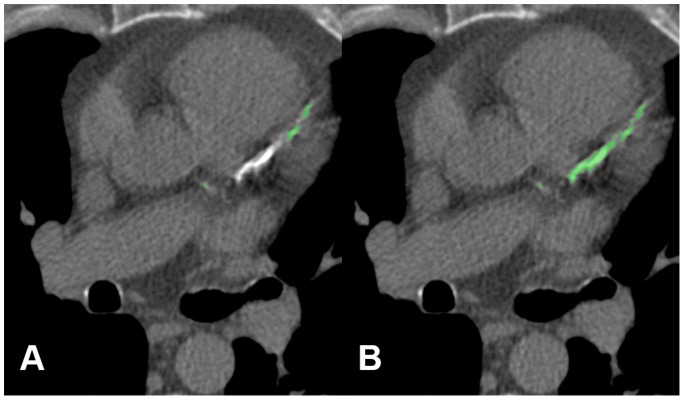
False negative by automated scoring. Example of an ‘outlier’ by automated quantification (A) compared to reference (B). In the LAD a severe calcification and black voids are visible. No stent was present.

**Figure 3:**
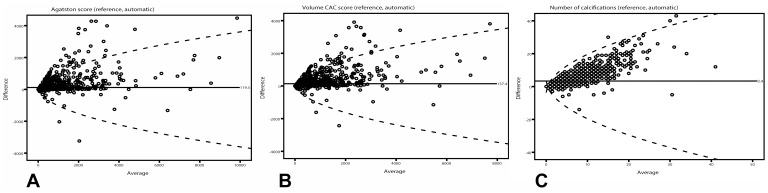
Bland-Altman plots of (A) Agatston, (B) volume, and (C) number of calcifications with 95% limits of agreement (dotted lines). Average score from reference and automated quantification is plotted against difference between the two quantification methods. The plots reveal underestimated calcium scores by automated quantification and an increasing difference with a higher average score. Regression formulas for absolute difference are multiplied by +/−1.96*(π/2)^0.5^ to get the 95% limits of agreement. For Agatston score: Y = (−64.482+15.332 *×^0.5^)*1.96*(π/2)^0.5^; For volume CAC score: Y = (−74.202+16.530*×^0.5^)*1.96*(π/2)^0.5^; and for number of calcifications: Y = (−1.743+3.073*×^0.5^)*1.96*(π/2)^0.5^.

The proportion of agreement between the Agatston strata of the reference and automated CAC score was 1386 (79.2%) of 1749 participants (Table 1). Further analysis of discordant pairs revealed that most discordant pairs occurred in the right coronary artery (RCA) and were due to unaccounted calcifications by the automated method (Table 2, Figure 1,2). A shift of more than one Agatston stratum was observed in 83 (4.7%). Bland-Altman plots (Figure 3) with the limits of agreement showing a systematic error due to an underestimation of automated quantified CAC scores and number of calcifications. Median difference was 2.5 (p25–p75∶0.0–53.2; QCD: 1.00) for Agatston score, 7.6 (p25–p75∶0.0–94.4; QCD: 1.00) for CAC volume and 1 (p25–p75∶0–5; QCD: 1.00) for number of calcifications.

**Table 1:** Agatston Risk Category Shift between reference and automated scores.

Reference Agatston score	Automated Agatston score					
	0	1–10	11–100	101–400	>400	Total
0	401	23	6	3	0	433
1–10	94	88	11	0	0	193
11–100	40	31	275	5	3	354
101–400	6	6	44	243	2	301
>400	4	1	14	70	379	468
Total	545	149	350	321	384	1749

**Table 2:** The coronary artery calcifications that were the main reason for discordance, based on Agatston risk category shift (n = 363).

Coronary region	Number (%)	False negative	False positive
Left Main	41 (11.3)	37	4
Left anterior descending	76 (20.9)	63	13
Left circumflex	95 (26.2)	74	21
Right coronary artery	125 (34.4)	111	14
Posterior descending	26 (7.2)	25	1

### Reliability of Reference and Automated CAC Score

For Agatston risk categories the linearly weighted kappa demonstrated very good reliability (κ = 0.85) [26]. For continuous values, despite underestimation CAC scores by automated quantification, the ICC was very good for Agatston score (0.90), very good for calcium volume (0.88) and good for number of calcifications (0.64).

### Human Interrater Reliability

**Figure 4:**
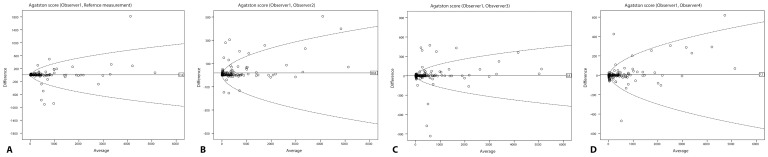
Bland-Altman plots of Agatston score with 95% limits of agreement (dotted lines) comparing the board certified chest radiologist (Observer1) with Reference (A), observer 2 (B), 3 (C), 4 (D).

Human interrater reliability was calculated based on a subset of 199 consecutive participants. Kendall’s w for Agatston risk categories among the four human raters was very good (0.88). The ICC among the four human observers was very good for Agatston score (0.95), for calcium volume (0.96) and for number of calcifications (0.89). The ICC between fully manual scoring and reference scoring was at least 0.96 for Agatston score, 0.97 of calcium volume and 0.90 for number of calcifications. Bland-Altman plots (Figure 4) with the limits of agreement compare the performance of board certified chest radiologist with the reference standard and with each observer.

## Discussion

This current study demonstrates that CAC score can be quantified on non-gated chest CT using automated software. The agreement and reliability of the fully automated scoring are good when compared to reference scores. Lung cancer screening for which guidelines have been published [27] enables additional identification of subjects at risk of CVD. Given the large number of potential participants automated quantification may prove of great value.

The application of CAC quantification with CT as a screening test has been proposed and adds incremental information for prediction of all-cause mortality and cardiovascular events [28], [29]. Moreover, lung cancer screening participants are at increased risk of a cardiovascular event, since aging and smoking are important risk factors for both conditions [30]. Automated quantification of CAC would allow cardiovascular risk stratification without additional costs and without additional radiation exposure for the participants. To employ automatic quantification of CAC, high agreement and reliability of the algorithm are very important for longitudinal follow-up and to guide treatment [31].

This study demonstrates good agreement and very good reliability of the evaluated algorithm. Nevertheless, errors were present and automatically obtained scores are systematically lower than those defined by the raters. However, comparison to interscan agreement in low dose, non-ECG synchronized chest scans reveals that the errors of the automatic scoring are similar to those that would be obtained by manual expert scoring in another scan [12]. Namely, the software incorrectly classified a calcium score of zero in 8.2% (144/1749). For a comparison, due to the interscan variation 5.3% (31/584) of scans had positive by the first and zero score by the second scan. [16] Furthermore, in our study a shift of more than one Agatston risk category was found in less than 5% of subjects. The majority of these shifts was in the risk categories with an Agatston score of less than 100. Scores higher than 100 are related to an increased atherosclerotic burden, multi-vessel disease, coronary heart disease and overall cardiovascular events [32], [33]. Previous research showed that the main causes of discordance are higher level of noise, motion artifacts and motion unsharpness congruent with cardiac motion on low-dose non-gated CT [34]. In particular visualization of the right coronary artery is known to be difficult because of motion artifacts [35]. In the present study we found a high prevalence of CAC, therefore we had enough power to assess agreement and reliability, since variability in CAC score is strongly linked with the total amount of CAC.

A recent meta-analysis determined the reliability between gated and non-gated CT and found a very good pooled Cohen’s kappa (κ = 0.89), however in the non-gated group the cardiovascular event rate was higher in subjects without CAC showing that is it not possible to exclude CAC on non-gated scans [11]. One previous study, in which Agatston scores were derived from non-gated chest CT scans, demonstrated good interscan reliability for Agatston risk categories (unweighted κ = 0.67) and very good interrater reliability (ICC = 0.97) [16]. The interrater reliability we observed in this study was only slightly less, which may be caused by the difference in experience between the raters. In line with previous research evaluating automated CAC scoring using non-gated CT we also observed an underestimation of CAC score [17].

Evaluation of CVD risk in lung cancer screening studies could also be performed manually in a semi-quantitative manner using ordinal scale. Such evaluation might relate well with CVD events [36]. However, such scoring would require expert time. This study demonstrates that fully automatic quantitative CAC scoring is feasible in large scale lung cancer screening trials without additional expert time.

Our study has several limitations. First, scans were obtained using low-dose non-gated chest CT, thus resulting in increased levels of noise and artifacts due to cardiac motion. However, this is current practice in lung cancer screening. Moreover, earlier studies demonstrated that coronary calcium scores determined with low-dose non-ECG synchronized chest CT correlate well with scores obtained with dedicated ECG-gated cardiac CT [14], [15] and that they are strong and independent predictor of cardiovascular events [10], [11]. Second, the reference standard for CAC was defined using a preliminary version of the automated software with subsequent manual correction. This made establishing of the reference standard easier and quicker, and thus made the study feasible in a large set of scans. However, the readers might have been biased by the presented results and therefore, we investigated whether this induced errors in the reference segmentations. The ICC between fully manual scoring and manually corrected reference results was very good (all >0.90), indicating the little effect of automatic presegmentation on the reference standard. Another limitation of our study was that manual scoring was performed partly by medical students. They however received intensive training for this study by a board certified chest radiologist, and in addition, independent reader inspected results of manual scoring and excluded scans with gross segmentation errors and metal artifacts. In patients with metal coronary stents calcium scoring would not result in risk reclassification. Also, in the remaining data set, the ICC between the four raters was very good. Finally, the method was evaluated with lung cancer screening scans acquired at single site. Future work will aim to broaden the evaluation of the method to scans acquired in multiple centers and possibly to scans made in multiple lung cancer screening trails.

In summary, automated quantification of CAC is feasible in non-gated non-contrast enhanced chest CT with good reliability and agreement when compared to reference scores. Nevertheless, CAC scores are lower when quantified automatically. The false negative zero scores indicate concern about the possibility to accurately identify subjects having a zero or low calcium score. The application of automated quantification of CAC in a lung cancer screening population can widen the scope of screening and help identify participants with a high-risk for cardiovascular events [37].
